# P130Cas/*bcar1* mediates zebrafish caudal vein plexus angiogenesis

**DOI:** 10.1038/s41598-020-71753-w

**Published:** 2020-09-24

**Authors:** Laura Wisniewski, Vanessa French, Nicola Lockwood, Leonardo E. Valdivia, Paul Frankel

**Affiliations:** 1 Institute of Cardiovascular Science, University College London, 5 University Street, London, WC1E 6JF UK; 2 Division of Medicine, University College London, 5 University Street, London, WC1E 6JF UK; 3 Center for Integrative Biology, Faculty of Sciences, Universidad Mayor, Santiago, Chile; 4 Present Address: Queen Mary University of London, London, EC1M 6BQ UK; 5 Present Address: The Francis Crick Institute, 1 Midland Road, London, NW1 1AT UK

**Keywords:** Angiogenesis, Embryogenesis, Developmental biology, Cell biology, Cell migration, Cell signalling

## Abstract

P130CAS/*BCAR1* belongs to the CAS family of adaptor proteins, with important regulatory roles in cell migration, cell cycle control, and apoptosis. Previously, we and others showed that P130CAS mediates VEGF-A and PDGF signalling in vitro, but its cardiovascular function in vivo remains relatively unexplored. We characterise here a novel deletion model of P130CAS in zebrafish. Using in vivo microscopy and transgenic vascular reporters, we observed that while *bcar1*−/− zebrafish showed no arterial angiogenic or heart defects during development, they strikingly failed to form the caudal vein plexus (CVP). Endothelial cells (ECs) within the CVP of *bcar1*−/− embryos produced fewer filopodial structures and did not detach efficiently from neighbouring cells, resulting in a significant reduction in ventral extension and overall CVP area. Mechanistically, we show that P130Cas mediates Bmp2b-induced ectopic angiogenic sprouting of ECs in the developing embryo and provide pharmacological evidence for a role of Src family kinases in CVP development.

## Introduction

Angiogenesis describes the formation of blood vessels from existing vessels and is the major mechanism of vascular growth. It is the predominant form of establishing and maintaining the complex blood vessels network which supports any vertebrate organism^1^. Arterial and venous specification is one of the early events of vascular development and is critical for establishing and maintaining a functioning vascular system^2^. It is a complex process largely determined by genetic factors and occurs independently of blood flow^3^. Genetic loss of arterial *vs* venous specification leads to arteriovenous malformations including A-V shunts that are characteristic of congenital arteriopathies such as hereditary haemorrhagic telangiectasia^2,4,5^. Many pathways regulating angiogenesis have been shown to be involved in the determination of arteriovenous cell identity, the best studied of which is the Vascular Endothelial Growth Factor (VEGF) signalling pathway. It plays a central role in arterial specification and differentiation, as well as being critical for the function and maintenance of vascular endothelium, e.g., by promoting endothelial cell (EC) proliferation and migration or increasing vascular permeability^6,7^. Venous specification is less well understood but signalling molecules such as EphB4, COUP-TFII, Neuropilin-2, Endomucin, Flt-4, and BMP have been implicated^3,8^. Recently, Bmp2-Bmpr2 signalling was shown to regulate venous cell migration in the caudal vein plexus of the developing zebrafish embryo^9^. Mechanistically, this was shown to be through downstream activation of the small GTPase Cdc42 and the actin binding protein Fmnl3^10^. However, how these signals are connected intracellularly remains unclear.

Members of the CAS (Crk-associated substrate) family of adaptor proteins have emerged as highly connected signalling nodes, with important regulatory roles in normal and pathological cell functions. Our group and others have shown that phosphorylation of P130CAS as a result of integrin-mediated adhesion, receptor tyrosine kinase or G-coupled receptor activity, hypoxia, mechanical stretching, or chemokine receptor activation enables different multi-protein complexes which impart downstream signalling, e.g., through FAK, CRK, DOCK180, 14-3-3, SRC, DOK1, ABL, RAP1, IQGAP1 or ERK, controlling cell attachment, migration, cell cycle progression, and cell survival signalling^11–13^. We previously showed that VEGF or Platelet Derived Growth Factor (PDGF) stimulation will induce migration and proliferation of endothelial or vascular smooth muscle cells, respectively, through P130CAS in vitro^12–15^. P130CAS was shown to be essential for the development of the cardiovascular system, as *Bcar1*−/− knockout mice are embryonic lethal at E11.5–12.5 with severe cardiovascular defects and poorly developed hearts^16^. Interestingly, the same group later reported that the specific deletion of the SH3 domain also caused embryonic death between E12.5 and 13.5. However, these animals did not present with cardiovascular complications but died due to a collapse of liver tissue because the sinusoidal ECs did not form fenestrae^17^. In contrast, no other in vivo model has reported lethality or commented on cardiovascular defects.

The zebrafish *Danio rerio* has gained significant popularity in recent years as an ideal model organism for studying angiogenesis in development and regeneration^18,19^. Zebrafish embryos can be kept translucent for 120 h post fertilisation (hpf) of development, which makes them highly suitable for live imaging. Many transgenic lines exist which label specific cell populations and key imaging modalities have been adapted or developed for use in zebrafish^20–22^. In zebrafish vascular development, vasculogenesis forms the dorsal aorta and posterior cardinal vein by 26hpf which is also the onset of circulation. All further blood vessels are formed via angiogenic sprouting, firstly arterial (from 26hpf onwards), followed by venous and lymphatic sprouting (from 36hpf onwards), and later coverage by perivascular cells (from 60hpf onwards). Analysis of the stereotypical embryonic vascular development allowed identification of key signalling pathways and in vivo observations of critical angiogenic concepts such as tip-and-stalk cell migration or perivascular cell coverage^22–25^.

We present here a novel knockout model of P130CAS in the zebrafish. Unlike the reported mouse model, *bcar1−/− *zebrafish showed no arterial angiogenic defects or heart defects during development; however, we observed a striking failure to form the caudal vein plexus. This vascular bed has been shown to be regulated by Bmp2b and angiogenic sprouting involves the actions of Cdc42 and Fmnl3^9,10^. We show here that inducible Bmp2b expression, leading to ectopic vessel formation in the developing embryo, is compromised in *bcar1−/− *zebrafish, suggesting that P130Cas is acting downstream of Bmp2b/Bmpr2a/b activation. We further present evidence that pharmacological inhibition of Src family kinases, the main kinases phosphorylating P130Cas^14^, leads to defective CVP formation in the developing embryo. We therefore propose that P130Cas is one of the key intracellular proteins allowing endothelial cells to recognise and respond differently to arterial and venous angiogenic cues.

## Results

### A deletion model for P130Cas/*bcar1* in zebrafish

Of the CAS proteins, only P130CAS is required for normal cardiovascular and embryonic development in mice^16,17,26^. However, the early lethality induced by P130CAS deletion has prevented more detailed mechanistic studies. The zebrafish genome harbours unique orthologues for all four CAS family proteins but there are no studies describing their functions to date. Sequence alignment of human and zebrafish P130CAS proteins shows an overall homology of 58.7% (see Supplemental Fig. S1A). The SH3 and C-terminal domains, as well as many of the tyrosine residues shown to be required for P130CAS activity, are particularly well conserved, with the SH3 domain being > 95% identical (see Supplemental Fig. S1). This suggests that the zebrafish P130Cas protein is likely to have comparable functions, making it a suitable model to study P130CAS function.

**Figure 1:**
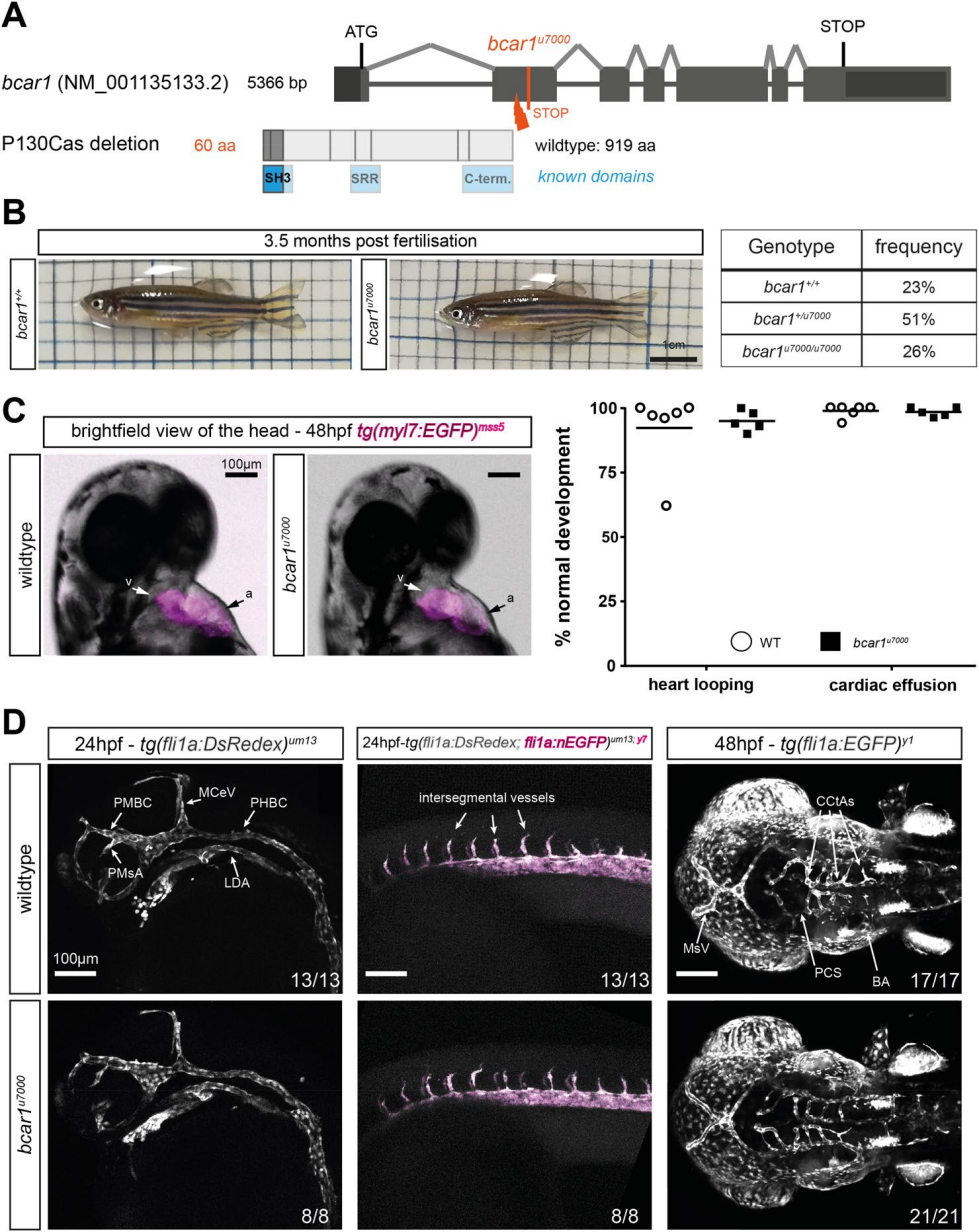
Loss of P130Cas does not induce lethality, major morphological defects, or defects in early arterial angiogenesis in zebrafish. (**A**) Schematic showing the exon–intron structure of *bcar1* and location of the genetic insertion-deletion of *bcar1*^*u7000*^ resulting in protein truncation during translation, known protein domains are indicated in blue. (**B**) Photographs of live adults (left) and diagramme showing actual observed viability from a *bcar1*^*u7000/*+^ heterozygous incross (right). Homozygous deleted fish are observed with expected Mendelian frequency; morphologically, they appear indistinguishable from wildtype siblings. (**C**) Brightfield image with fluorescent overlay of *tg(myl7:EGFP)*^*mss5*^ zebrafish hearts at 48hpf (left), clearly showing ventricle (v) and atrium (a). Quantification of n = 6 wildtype and n = 5 *bcar1*^*u7000*^ clutches with n ≥ 53 embryos each (right) confirm the absence of heart looping or effusion (oedema) defects at 48hpf. (**D**) Maximum intensity projections, lateral views of the head (left column) and trunk (middle column) at 24hpf shows emergence of primordial midbrain channel (PMBC), middle cerebral vein (MCeV), primordial hindbrain channel (PHBC), lateral dorsal aorta (LDA), primitive mesencephalic artery (PMsA), and intersegmental vessel sprouting as normal in *bcar1*^*u7000*^ mutants. Dorsal to ventral view shows emergence of cerebral central arteries (CCtAs), basilar artery (BA) and posterior communicating segment (PCS) by 48hpf as normal (right column). Experimental *n* as indicated per image, from ≥ 2 independent clutches. Scale bars as indicated.

In order to investigate the cardiovascular function of P130Cas in zebrafish, we generated a global deletion model of *bcar1* using CRISPR/Cas9 gene editing, targeting the second exon of *bcar1*, which encodes the SH3 domain (see Fig. 1A). We identified fish harbouring a frameshift mutation that results in a truncated protein of 60 amino acids, with predicted loss of all functional domains (designated allele *bcar1*^*u7000*^). We confirmed protein loss in embryos and adults, both using immunoblotting and whole mount immunofluorescence staining (see Supplemental Fig. S2). Immunofluorescence revealed a ubiquitous staining pattern, suggesting that P130Cas is expressed homogenously throughout the zebrafish embryo; this is in line with the literature for rodent P130CAS (see Supplemental Fig. S2B)^27^.

### P130Cas is dispensable for cardiac development, early VEGF-induced angiogenesis, and vasculogenesis

Surprisingly, unlike the mouse model, *bcar1*^*u7000*^ embryos and adults were obtained with expected Mendelian frequency, showed similar survival rates during juvenile development and adulthood, and appeared visually indistinguishable from wildtype or heterozygous siblings (see Fig. 1B, Supplementary Fig. S3A,C). We raised maternal zygotic mutants and found slight but statistically significant differences in size between WT and *bcar1*^*u7000*^ embryos and adults (Supplementary Fig. S3B). Whilst *bcar1*^*u7000*^ embryos and adults were smaller, the biological significance of this minor size difference remains to be determined.

A genome-wide RNA tomography sequencing study identified *bcar1* transcript at 10- and 15-somite stage^28^. Immunoblotting of total embryo lysates confirmed P130Cas protein expression at 24hpf, 30hpf, and 48hpf, as well as in the adult (see Supplemental Fig. S2A). These findings would support a potential role for P130Cas in cardiovascular development.

Because the P130CAS deletion mouse model dies in utero of cardiac pump failure^16^, we first investigated the cardiac development of zebrafish embryos. To identify potential defects, we studied embryonic heart rate, morphological heart formation, and blood flow dynamics of *bcar1*^*u7000*^ embryos. We did not observe heart looping or cardiac effusion defects, which are typical phenotypes associated with genes involved in heart organogenesis (see Fig. 1C). The heart rate of *bcar1*^*u7000*^ embryos was slightly increased in comparison to wildtype (mean difference of 5.51%, *p* = 0.046, see Supplemental Fig. S4D). However, we did not observe cardiac pump failure as described in the mouse model, suggesting that P130Cas is not required for cardiomyocyte contractility in the zebrafish during development.

We did not detect any morphological differences between homozygous mutant and wildtype embryos during key stages of cardiac organogenesis using brightfield and fluorescence imaging of the developing heart with *tg(myl7:EGFP)*^*mss5*^ transgenic fish (see Fig. 1C). In line with this normal cardiac development, the onset of blood flow and overall flow patterns, e.g., the establishment of the first circulatory loop by 24hpf or the looping from dorsal aorta into posterior cardinal vein in the caudal tissue were also unaffected in *bcar1*^*u7000*^ embryos (see Supplemental Fig. S4B, data not shown for onset of flow). Taken together, these results suggest that P130Cas is not required for cardiac organogenesis or the establishment of circulation in the developing zebrafish.

Work by our group and others has described that P130CAS plays an important role in VEGF-A- and NRP1-mediated endothelial cell (EC) signalling and angiogenesis in vitro^13,15,29^. Because early arterial angiogenesis and vasculogenesis in zebrafish fundamentally require Vegfaa/ab signalling^9,30,31^, we expected *bcar1*^*u7000*^ embryos to show vascular defects during early development. To our surprise, we observed normal vasculogenesis and arterial angiogenesis. These included the sprouting of arterial intersegmental vessels and prominent cerebral vessels such as the basilar artery and cerebral central arteries, as well as formation of the primary vessels formed via vasculogenesis such as the lateral dorsal aorta and posterior cardinal vein (see Fig. 1D).

### P130Cas is required for correct development of the caudal vein plexus

**Figure 2:**
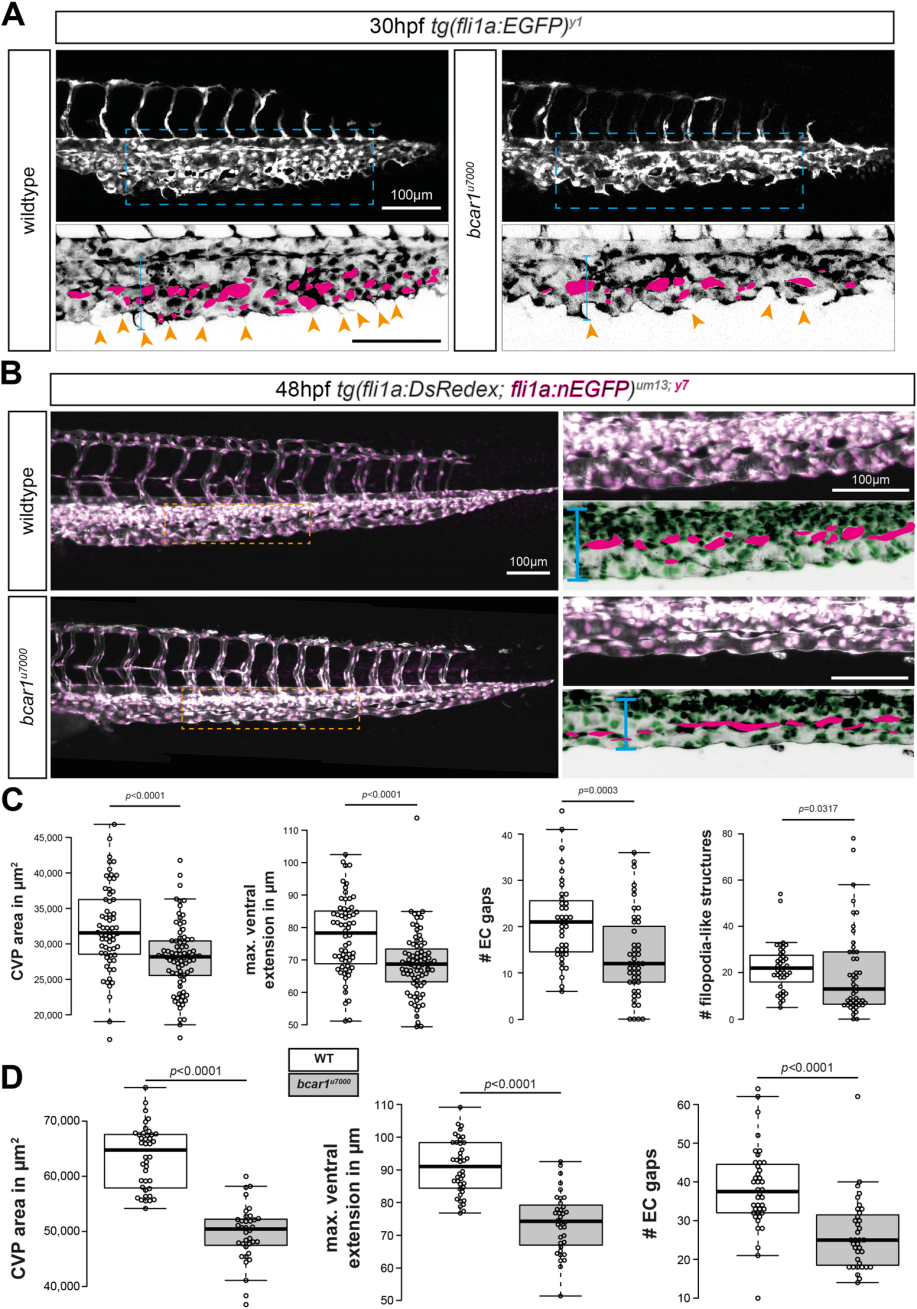
P130Cas is required for caudal vein plexus (CVP) angiogenesis. (**A**,**B**) Maximum intensity projections of wildtype and *bcar1*^*u7000*^ embryos at 30hpf (**A**) and 48hpf (**B**), with inset (blue/orange dashed boxes) showing the sprouting front. Insets are inverted for easier visualisation. Blue line indicates ventral extension, magenta circles endothelial cell gaps, orange arrowheads filopodia-like cell protrusions. (**C**,**D**) Quantification of total CVP area, maximal ventral extension, and total non-vascularised area (= ‘EC gaps’) show a significant reduction at both time points. All results displayed as box plots with centre line showing median and box dimensions indicating 25th and 75th quartile. Each data point represents an individual embryo, experimental *n* from ≥ 3 independent clutches, *p* values as shown. White boxes are WT, light grey boxes indicate *bcar1*^*u7000*^. Scale bars as indicated.

To assess if P130Cas mediates angiogenic sprouting which is not primarily Vegfaa/ab-induced, we next investigated the development of the caudal vein plexus, the most posterior vascular bed in the developing embryo. As previously described, venous cells from the posterior cardinal vein will begin sprouting and actively migrating ventrally from approximately 24hpf onwards, to form a complex vascular network by ~ 32hpf, which remodels into a singular vein through regression of the dorsal posterior cardinal vein^10,32,33^. As shown in Fig. 2A, homozygous *bcar1*^*u7000*^ embryos failed to form and remodel this network properly: venous endothelial cells did not migrate as efficiently and did not detach from their neighbouring cells to allow for the semi-single-cell migration mode displayed by wildtype embryos. Instead, venous ECs lacking functional P130Cas remained in close contact and formed a thick venous tube or bulging stalks. They also formed fewer filopodia-like cell protrusions. This resulted in a narrower caudal vein plexus with a significantly reduced ventral expansion, reduced total vessel area, and reduced number of spaces between migrating endothelial cells (‘EC gaps’) (see Fig. 2C). The caudal tissue in general was not affected by lack of P130Cas, suggesting that this defect is a unique vascular requirement independent of other cell types (see Supplemental Fig. S4A,C).

Importantly, *bcar1*^*u7000*^ embryos did not fail to form the CVP because of a lack of endothelial cell proliferation. Both at 24hpf and 30hpf, i.e., before the onset of and during active angiogenic sprouting and migration, there was no difference in endothelial cell number, as assessed using the *fli1a:nEGFP*^*y7*^ transgene (see Supplementary Fig. S5A). This suggests that P130Cas is specifically required for EC migration.

**Figure 3:**
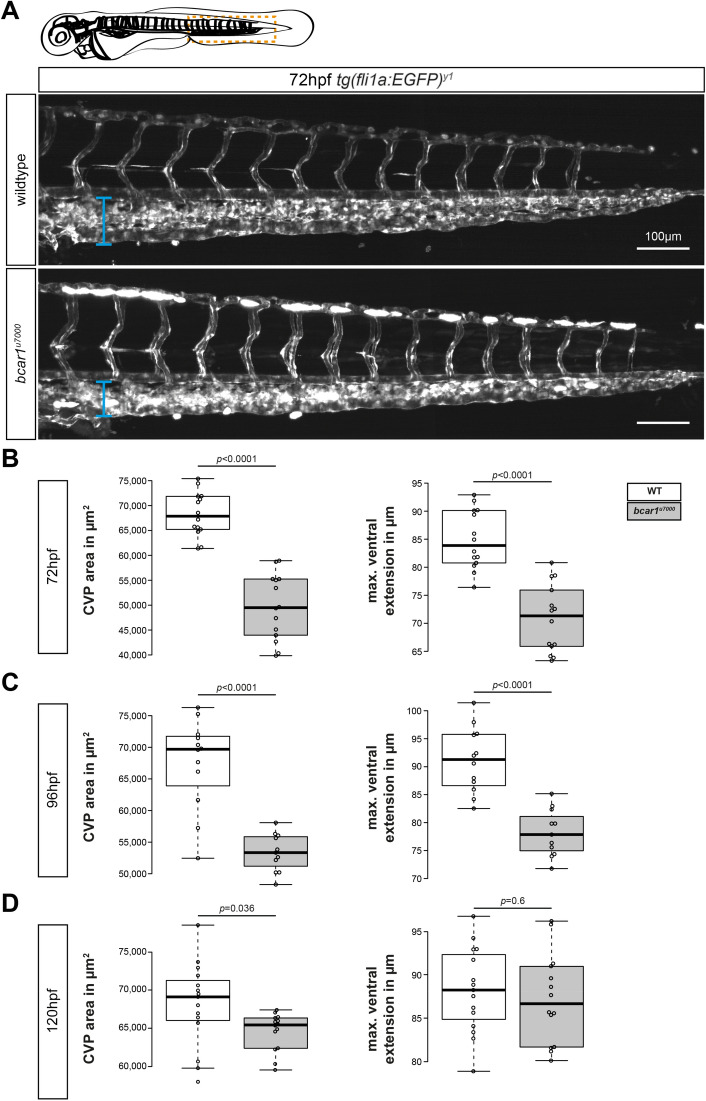
P130Cas deletion results in prolonged reduction of caudal vein plexus area. (**A**) Maximum intensity projections of the caudal vasculature of WT and *bcar1*^*u7000*^ embryos at 72hpf, from the anal pore, as illustrated by orange box in cartoon of embryo at the top. (**B–D**) Quantification of the maximum ventral extension of the CVP to the dorsal aorta, as well as overall vascular CVP area confirm that significant reductions remain in mutant embryos at 3dpf (**B**) and 4dpf (**C**). At 5dpf, the caudal area is still smaller but the ventral extension no longer significantly different (**D**). All results displayed as box plots with centre line showing median and box dimensions indicating 25th and 75th quartile. Each data point represents an individual embryo, experimental *n* from ≥ 2 independent clutches, *p* values as shown. White boxes are wildtype, light grey boxes indicate *bcar1*^*u7000*^. Scale bar as indicated.

We next asked if a lack of P130Cas merely causes a delay in caudal vein plexus formation. If this were the case, we would expect to observe full outgrowth of the plexus at a later time point and no differences at later stages of remodelling. However, at 48hpf, the compromised caudal vein plexus of homozygous *bcar1*^*u7000*^ embryos had not resolved (see Fig. 2B). Although a continuous vein was formed, it was located closer to the dorsal aorta than appropriate; the overall area of the caudal vein, as well as the number of spaces between endothelial stalks remained significantly reduced (see Fig. 2D). Even at 3 or 4dpf, *bcar1*^*u7000*^ embryos showed a persistent, significantly reduced vascularised area and the posterior vein remained significantly closer to the dorsal aorta (see Fig. 3). At 5dpf, the furthest distance between the posterior vein and dorsal aorta in the caudal region was unchanged but the overall caudal vein area remained significantly smaller (see Fig. 3D). Together, these results suggest that the endothelial cells of the CVP require P130Cas to migrate efficiently and that there are no compensatory mechanisms in place to replace this function.

### P130Cas is required for early lymphangiogenesis

During zebrafish development, there is a secondary wave of sprouting originating from the posterior cardinal vein, which begins shortly after CVP formation. These sprouts migrate dorsally to either connect to existing arterial intersegmental vessels (aISV) and turn these into venous intersegmental vessels (vISV), or to form the parachordal lymphangioblasts after acquiring lymphatic cell identity^34–36^. Interestingly, *bcar1*^*u7000*^ embryos had developed significantly fewer parachordal lymphangioblasts at 48hpf and these did not migrate across the somite, as in wildtype embryos (Supplementary Fig. S5B,C). By 5dpf, the number of lymphatic vessels such as the thoracic duct was reduced but the vessels were present (see Supplementary Fig. S5D,E), suggesting that P130Cas does not regulate the acquisition or maintenance of lymphatic endothelial cell identity per se but appears to play a role in lymphatic endothelial cell migration.

**Figure 4:**
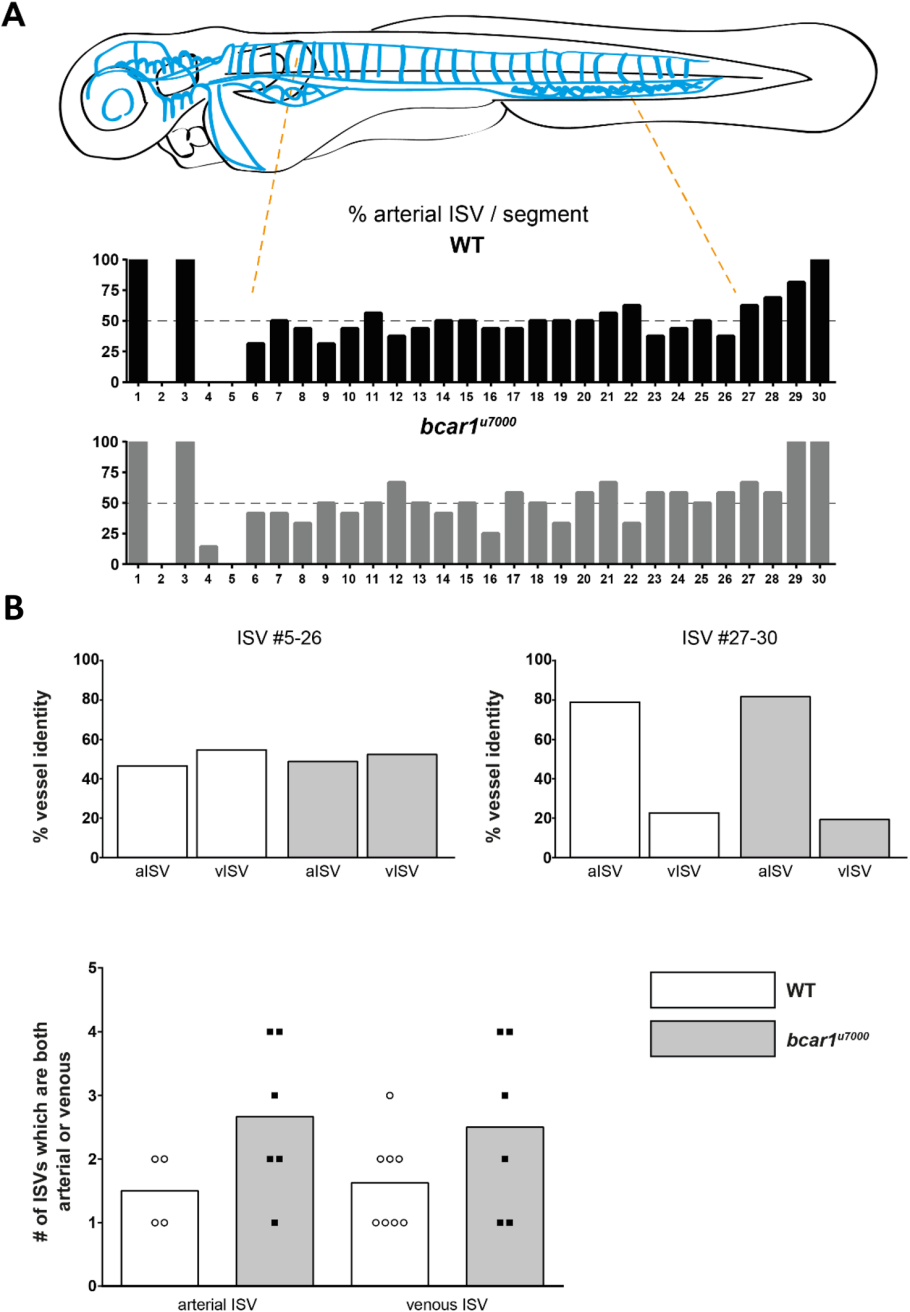
P130Cas is not required for venous intersegmental vessel formation at 72hpf. (**A**) Illustration showing the vasculature of the trunk in a stylised embryo, with bright blue symbolising arteries and dark blue veins. Bar graphs showing the percentage of ISVs which were arterial for wildtype (black) and *bcar1*^*u7000*^ (grey), from anterior to posterior. (**B**) Quantification of vessel identity for ISV pairs 5–26 ( left) or the last, more arterial, pairs 27–30 (right) did not show differences. A non-significant increase in the number of ISVs where both vessels were arterial or venous (bottom graph) was observed. White boxes indicate wildtype, grey boxes *bcar1*^*u7000*^.

Arterial and venous identity is acquired at random with the exception of the first five anterior intersegmental vessels and a preference towards aISV at the posterior end^34^. Importantly, analysis of the vasculature at 72hpf showed no difference in the number or distribution of arterial *vs* venous intersegmental vessels (see Fig. 4A). Neither did we observe any change in the identity of the first anterior vessels, *bcar1*^*u7000*^ embryos also showed predominantly aISV posteriorly (see Fig. 4B). We did observe a slight increase in the number of ISV pairs where both left and right vessel were of identical identity, i.e., either both connected to the dorsal aorta or both connected to the posterior cardinal vein but this was not significant (see Fig. 4B bottom panel). Taken together, these results indicate that P130Cas contributes to, but is not required for, dorsal migration of venous cells originating from the PCV. Similarly, P130Cas contributes to, but is not required for, efficient lymphatic endothelial cell migration. However, P130Cas is required for caudal vein plexus sprouting and remodelling. In agreement with a role for *bcar1* in CVP development, immunofluorescence staining demonstrated ubiquitous P130Cas expression in the CVP at 30hpf (see Supplementary Fig. S2B).

### P130Cas does not mediate general venous angiogenesis in the zebrafish embryo

We next asked if P130Cas might more broadly transduce signals promoting venous endothelial migration. To assess this, we investigated the formation of the subintestinal vein plexus (SIV). Angiogenesis in the SIV has been shown to be regulated by both Vegfa and Bmp signalling, with Vegfa regulating initial sprouting and proliferation and Bmp promoting plexus outgrowth^37,38^. Further, the endothelial cells forming the SIV also originate from the posterior cardinal vein, the same vein which gives rise to the caudal vein plexus^38^. Interestingly, we did not observe any vascular defects in this vascular bed, with *bcar1*^*u7000*^ embryos showing the normal basket pattern and no difference in outgrowth, or excessive/absent sprouting as observed in the literature (see Supplemental Fig. S6). This suggests that P130Cas does not mediate general venous sprouting but rather participates in a signalling pathway unique to the caudal vein plexus.

### P130Cas/bcar1 deletion blocks Bmp2b-induced formation of ectopic vessels

It has previously been shown that *bmp2b*, *bmpr2a*, and *bmpr2b* mRNA are specifically expressed in the caudal tissue of the developing embryo and that Bmp2b binding to its receptors Bmpr2a/2b induces angiogenic sprouting of venous cells from the posterior cardinal vein^9,10^. This led us to hypothesise that P130Cas is able to mediate Bmp2b-Bmpr2a/b signalling in venous sprouting.

**Figure 5:**
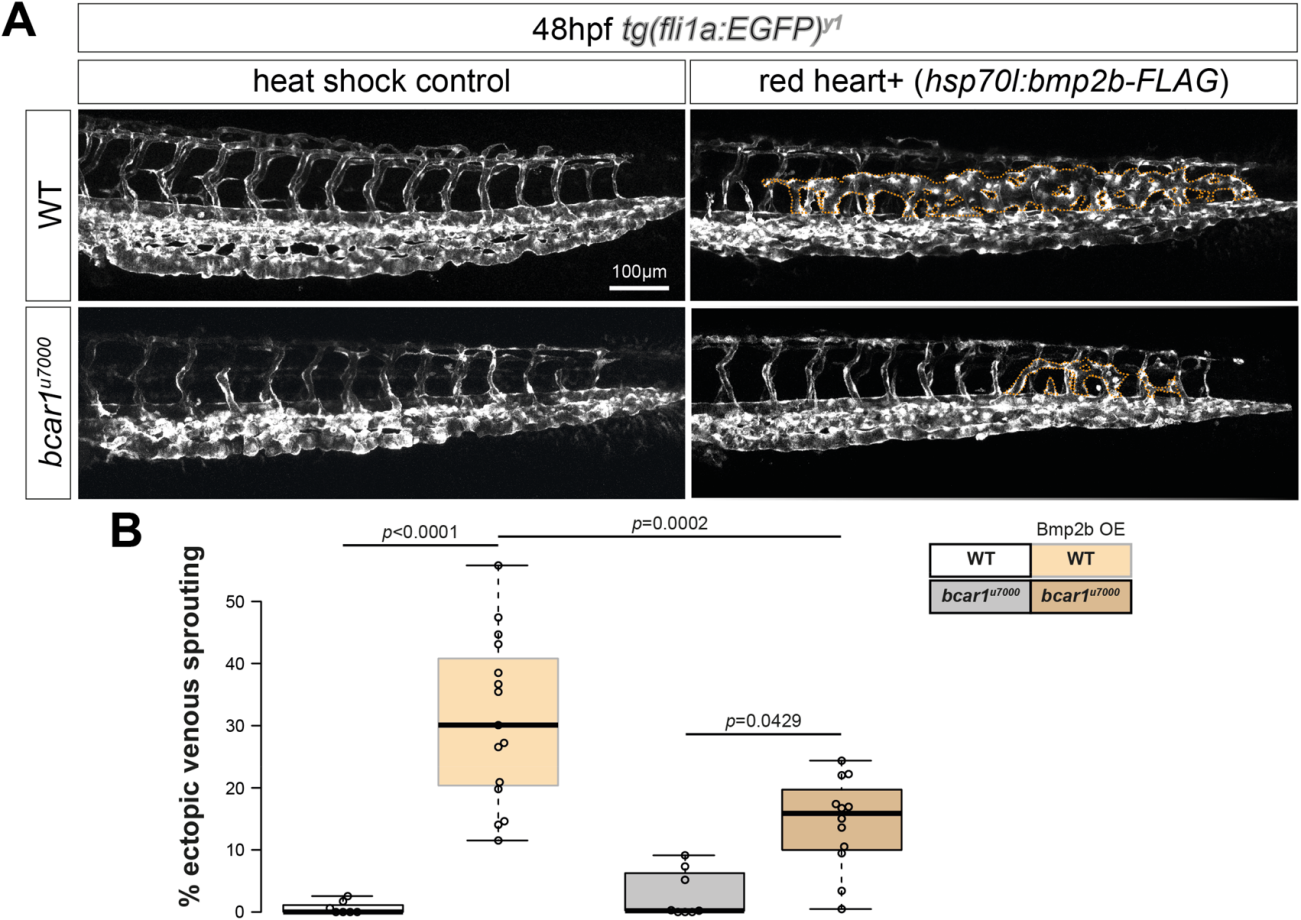
P130Cas mediates Bmp2b-induced formation of ectopic vessels. (**A**) Maximum intensity projections of WT and *bcar1*^*u7000*^ embryos, injected with *hsp70-bmp2b* plasmid and *tol2* transposase RNA and subjected to heat shock from 22 to 24hpf. Ectopic venous sprouting above the dorsal aorta (indicated by dotted orange lines) is induced by overexpression of Bmp2b (upper row). (**B**) Quantification showed a 50% reduction of ectopic sprouting in *bcar1*^*u7000*^ embryos (lower row). Data displayed as box plots with centre line showing median and box dimensions indicating 25th and 75th quartile. Each data point represents an individual embryo, experimental *n* from ≥ 3 independent experiments, *p* values as shown. White box indicates wildtype, orange box *bcar1*^*u7000*^ embryos, grey shaded boxes indicate *hsp70-bmp2b* plasmid integration. Scale bar as indicated.

To test for genetic interaction between P130Cas and Bmpr2a/b in CVP formation, we investigated whether P130Cas acts downstream from Bmp2b to regulate CVP formation. It is known that the overexpression of Bmp2b under a heat shock controlled promoter results in dorsal sprouting of ECs from the CVP, leading to the formation of ectopic venous vessels. Heat shock inducible expression of *bmp2b* at 24hpf in wildtype embryos caused the formation of ectopic vessels, whereas this was significantly reduced in *bcar1*^*u7000*^ embryos (see Fig. 5). This result provides strong evidence that P130Cas transduces Bmp2b-Bmpr2a/b signalling in venous endothelial cells during angiogenic sprouting of the caudal vein plexus.

### Pharmacological inhibition of Src family kinases leads to defective CVP formation

We next asked which protein transduces Bmp2b-Bmpr2 activation to P130Cas. It is known that Src family kinases (SFKs), including SRC, directly phosphorylate P130CAS at focal adhesions within a macromolecular complex required for endothelial cell migration in vitro^13,39^. It has also been reported that c-SRC directly binds the C-terminus of BMPR-II^40^.

**Figure 6:**
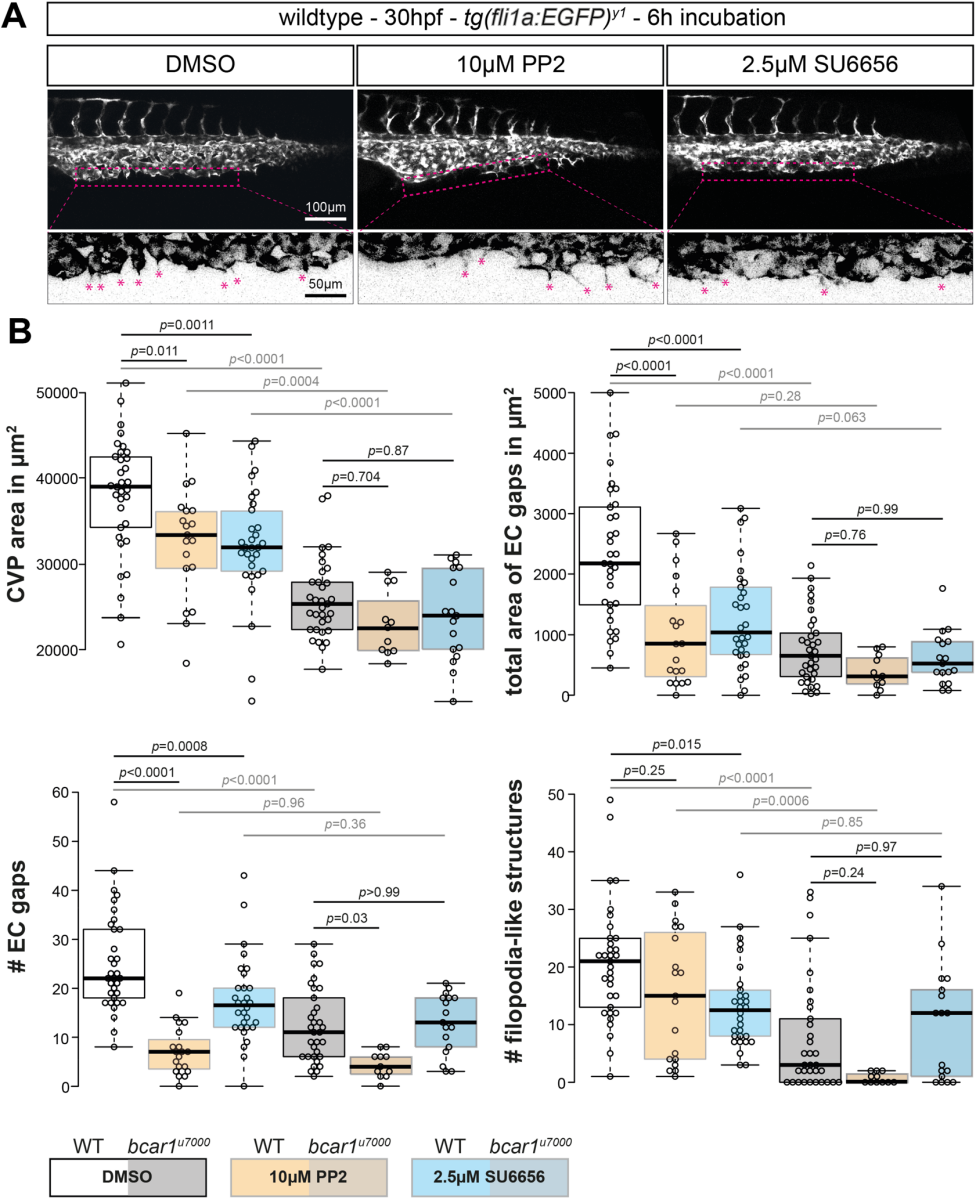
Src family kinases are required for caudal vein plexus formation. (**A**) Maximum intensity projections of wildtype embryos at 30hpf, exposed to DMSO control, PP2, or SU6656 inhibitors. Inhibition of Src family kinases caused defective CVP sprouting. Magenta dashed lines indicate the area of vascular front, shown below at higher magnification. Areas were inverted and contrast increased in FIJI for better visualisation. Magenta asterisks highlight filopodia-like protrusions. (**B**) Quantification of CVP area, number of endothelial gaps, total area of endothelial gaps, and number of filopodia-like cell protrusions showed significant reductions after inhibitor but not DMSO treatment. All results displayed as box plots with centre line showing median and box dimensions indicating 25th and 75th quartile. Each data point represents an individual embryo, experimental *n* from ≥ 3 independent experiments except for *bcar1*^*u7000*^ PP2 which is n = 1, *p* values as shown. Scale bars as indicated.

We therefore investigated if SFKs are also necessary for correct migration of venous endothelial cells forming the CVP in vivo. We treated zebrafish embryos with two chemically distinct and well known pharmacological inhibitors of SFKs, SU6656 and PP2^41,42^, and examined their effects on the development of the CVP. Inhibitors were added at the onset of caudal vein plexus sprouting (24hpf), to circumvent the requirement for the SFKs Fyn and Yes during gastrulation of zebrafish^43,44^. We observed that SFK inhibition impaired CVP formation similarly to loss of P130Cas, i.e., caudal vein plexus vessel area, number of filopodia-like extensions, and the area created by gaps between endothelial cells were significantly reduced in PP2- and SU6656-compared to vehicle DMSO-treated embryos. In *bcar1*^*u7000*^ embryos, inhibitor treatment did not significantly affect these parameters (see Fig. 6).

These results suggest that SFKs are required for efficient migration of venous endothelial cells in this vascular bed. The similarity in phenotype between drug treated and *bcar1*^*u7000*^ embryos, together with the known literature, support the conclusion that SFKs act upstream of P130Cas in caudal vein plexus formation.

## Discussion

Angiogenesis, the physiological process through which new blood vessels form from pre-existing vessels, is essential for development and in disease. The distinction between arteries and veins is one of the earliest and most important events of blood vessel development. We present here the first functional characterisation of an in vivo P130Cas global deletion model since the initial description of the *Bcar1*−/− knockout mouse^16^ and the first description of P130Cas activity in the zebrafish.

In line with reports from the literature showing that P130CAS is expressed in all analysed tissues in rodents^27^, we find ubiquitous expression of P130Cas in the embryo. In stark difference to the mouse, P130Cas-deleted zebrafish are viable and fertile. Importantly, this is not a unique occurrence and there are other genetic knockout zebrafish models which do not replicate lethality shown from global knockout in the mouse, e.g., the loss of *endoglin* or *wtx* are well tolerated by fish^5,45^. This has been ascribed to the robustness of the fish and functional compensation by other pathways but has crucially allowed functional insights into these proteins which are not possible using mice models. In line with this reasoning, deletion of the single CAS family protein in *Drosophila melanogaster* only marginally compromises viability but has no further effects, unless other signalling pathway components are also genetically deleted^46^. In mice, deletion of *Bcar1*^16,17^ results in severe cardiovascular defects, causing embryonic lethality. While we did not explicitly investigate if P130Cas was expressed in the heart during development, we did not observe any defects in gross heart morphology or function, nor lethality as shown in Fig. 1. These observations argue against a developmental requirement for P130Cas in the zebrafish heart.

One explanation for this discrepancy in phenotype is the well established fact that zebrafish embryos do not require a functional heart beat or indeed circulation for up to 6dpf^47^. In addition, our unpublished tissue-specific deletion studies of P130CAS in mouse development have shown that P130CAS appears primarily responsible for heart septation^48^. Septation does not occur in zebrafish and P130Cas is therefore not required to mediate this process, bypassing this function in the mouse^49^.

Zebrafish embryos lacking P130Cas did not show any vasculogenic or arterial angiogenic defects within the first 48hpf of development. This was surprising as all the available in vitro literature strongly implies that P130CAS mediates VEGF-A activity and most early angiogenic sprouting in zebrafish is strongly dependent on Vegfaa/b signalling^9,14,31^. These results therefore suggest that P130Cas is not required for these processes in vivo and may not mediate Vegfa signalling in zebrafish. This could be explained by evolutionary functional divergence between fish and higher vertebrates, although it does not exclude the possibility that P130Cas contributes to Vegfa-induced angiogenesis, either during embryogenesis or adulthood. However, further studies are needed to address this.

We show here a critical role for P130Cas in sprouting venous angiogenesis leading to CVP formation in the developing zebrafish embryo. Defects in homozygous deleted animals include reductions in CVP area, maximal ventral extension on the CVP, EC gaps, and filopodia like extensions.

Sprouting and migration in this vascular bed have been shown to be regulated by Bmp2b activity through Bmpr2a/b and a downstream cascade involving Arhgef9b-Cdc42-Fmnl3, enabling filopodia formation^9,10^. Filopodia formation is considered important for angiogenic sprouting of tip cells, although in the zebrafish trunk the intersegmental vessels are able to complete migration even in the absence of filopodia^50^. However, in the caudal vein plexus, abrogation of filopodia critically impairs migration efficiency and results in a dense vascular plexus^10^.

The similarity in phenotype between embryos deficient for *bmpr2a/b* and *bcar1*^*u7000*^ embryos suggest that P130Cas could play a novel role in Bmp2b signalling required for CVP angiogenesis. Genetic overexpression of the Bmp2b ligand causes ectopic venous sprouting into the somatic tissue dorsal of the dorsal aorta^9^. Consistent with these data and our results presented here, inducible overexpression of Bmp2b resulted in ectopic vessel production in wildtype embryos, whereas *bcar1*^*u7000*^ embryos showed 50% less ectopic sprouting. Furthermore, we show that pharmacological inhibition of SFKs during CVP formation phenocopied the *bcar1*^*u7000*^ mutants. It is well established that SFKs play an important role in regulating P130CAS tyrosine phosphorylation and downstream function^14^. Of interest, a yeast two-hybrid screen identified c-SRC as a direct binding partner of BMPR-II which was confirmed in a co-immunoprecipitation experiment in HEK293 cells^40^. Furthermore, a recent study found SRC activity to be required for BMP2-induced cell motility and actin polymerisation in mouse epicardial cells^51^. Future studies should aim to further define the mechanistic relationship between SFKs and P130Cas in CVP formation. While we here provide some evidence indicating that SFKs could mediate this interaction, further studies are required to confirm this pathway, such as decreased tyrosine phosphorylation of P130Cas in response to SFK inhibitor treatment of embryos. These studies will reveal a more detailed understanding of the upstream and downstream signalling pathways involved in P130Cas mediated CVP formation, including its potential role in the (non)canonical BMP2 signalling pathways.

P130CAS has been identified as regulating migration and enabling invasion via transcriptional upregulation of Matrix Metalloproteinases (MMPs), required for degradation of the extracellular matrix^52,53^. It is also a key player for filopodia formation, especially in the filopodial tip, and absence of P130CAS causes filopodia to become unstable^54^. Intriguingly, there is also evidence linking the known P130CAS effector IQGAP1 to ARHGEF9-CDC42 signalling across several cell lines^55^. Further supporting this potential downstream pathway, in our recent proteomic interactor screen, where human umbilical vein endothelial cells were stimulated with VEGF and proteins bound to P130CAS were analysed by mass spectrometry, both CDC42 Binding Protein and FMNL3 were detected as binding partners^29^.

Taken together, our results show that in zebrafish, P130Cas plays an important role in mediating CVP angiogenesis, and suggests a novel role for P130Cas in Bmp2 signalling required for venous angiogenesis.

## Methods

### Key resources table

**Table:** 

Reagent type or resource	Designation	Source or reference	Identifiers	Additional comments
Strain (*Danio rerio*)	Fli1a:EGFP^y1^	PMID: 12167406		
Strain (Danio rerio)	Fli1a:nEGFP^y7^	PMID: 12050147		Donated by Jason Rihel, UCL Biosciences
Strain (Danio rerio)	Fli1a:DsRedex^um13^	PMID: 19269286		Donated by Nadia Mercader, University of Bern
Strain (Danio rerio)	Flt4BAC:mCitrine^hu7135^	PMID: 24523456		Donated by Jason Rihel, UCL Biosciences
Strain (Danio rerio)	Myl7:EGFP^mss5^	PMID: 24777450		Donated by Caroline Pellet-Many, RVC
Antibody	Anti-P130Cas (mouse)	BD Biosciences	#610271	
	Anti-beta-tubulin (mouse)	Santa Cruz	Sc-55529	
	Anti-mouse (horse)	Cell Signaling Technology	#7076S	
	Alexa-Fluor 488 anti-mouse (rabbit)	invitrogen	#A-21204	
Commercial assay, kit	QIAquick PCR purification kit	QIAGEN	28004	
	HiScribe T7 High Yield RNA synthesis kit	NEB	E2040S	
	RNeasy Mini Kit	QIAGEN	74104	
	T3 mMessage mMachine	Ambion	AM1348	
	RNA Clean & Concentrator-5	Zymo	R1013	
	Precision Melt Supermix	BioRad	1725110	
	Cas9 plasmid			Donated by Steve Wilson, UCL Biosciences
Chemical compound, drug	PTU (n-phenylthiourea)	Sigma	P7629	PubChem 57654544
	PP2	tocris	#1407	PubChem 4878
	SU6656	tocris	#6,475	PubChem 24278818
Software, algorithm	FIJI		https://fiji.sc/	
	GraphPad Prism		https://www.graphpad.com/scientific-software/prism/	
	BoxPlotR		https://shiny.chemgrid.org/boxplotr/	

### *bcar1*^*u7000*^ line generation/CRISPRCas9 transgenesis

Gene-specific guide RNA sequence for *bcar1* was as follows.

#### GTGCTGGAGCGGGACACGCA

Guide RNA was synthesised according to the protocol described by^56^, via transcription from a double stranded DNA, and purified using the RNeasy Mini kit (QIAGEN). The sequence was targeted to exon 2 to disrupt protein function as early as possible, and designed using https://crispr.mit.edu, developed by^57^. The oligonucleotides were synthesised by Eurofins Genomics (Germany). Wildtype embryos were injected at one-cell-stage with 150 pg *bcar1* guideRNA and 15 pg *cas9* mRNA. HRMA confirmed that co-injected embryos showed indels in the *bcar1* locus. F0 Adults were screened for genetic transmission and the *bcar1*^*u7000*^ line established after Sanger sequencing of F1 adult tissue samples. The u7000 allele has a 2 bp indel which results in a premature stop codon at position 61.

### Heat shock and drug inhibition

To overexpress Bmp2b, embryos were injected at one-cell-stage with 25 pg *tol2* transposase mRNA and 12.5 pg *pTol2-cmlc2:NLS-mCherry-HS4-hsp70l:bmp2b-FLAG* plasmid (kindly donated by Naoki Mochizuki, NCVC, Osaka, Japan)^9,10^. To induce heat shock, embryos were transferred into warmed 39 °C E3 medium and placed into a 39 °C incubator for 1 h from 24hpf. After this time, medium was replaced by standard 28.5 °C E3 medium and dishes returned to a standard incubator set at 28.5 °C. Embryos showing nuclear mCherry expression in cardiomyocytes were considered to be positive for Bmp2b overexpression.

For drug inhibition experiments, embryos were dechorionated and placed into E3 medium containing 1–25 µM PP2 or 1–10 µM SU6656, or the equivalent volume of DMSO, from 24 to 30hpf.

### Protein extraction from zebrafish tissue

Freshly extracted samples were placed into 50 µl RIPA buffer (R0278, sigma-aldrich, MO, USA) containing 1:1,000 phosphatase inhibitor cocktails 1 and 2 (P2850, P5726, sigma-aldrich) and 1X protease inhibitor cocktail (cOmplete Mini, EDTA-free, sigma-aldrich). Samples were homogenised using a hand-held motorised homogeniser with individual sterile plastic pestles (PELLET PESTLE cordless motor, 749540-0000, and pestles, 749521-1500, Kimble Chase, DWK Life Sciences LLC, Wertheim/Main, Germany), then debris pelleted by centrifugation at > 12,000×*g* for 10 min at 4 °C. 37.5 µl supernatant containing protein was transferred into a new Eppendorf tube containing 12.5 µl 4 × SDS Sample Buffer (novagen, EMD Millipore Corp, MA, USA). Samples were denatured by boiling at 95 °C for 5 min and stored at − 20 °C. Embryo samples were typically pooled, with ~ 2–3 embryos yielding 1 sample, while individual fin tissue samples yielded sufficient protein.

### Immunoblotting

Western Blot was performed as per standard conditions and as previously described, e.g., by^58^, using NuPAGE Bis–Tris gels and associated buffer solutions (Invitrogen, now Thermo Fisher Scientific). Briefly, approximately 15-20 µl protein lysates and Spectra BR protein ladder (Fermentas, #26623) were loaded into 4–12% Bis–Tris gels and separated for ~ 1 h at 200 V in NuPAGE MOPS running buffer. Gels were transferred in NuPAGE Transfer buffer, containing 10% methanol, onto methanol-activated PVDF membranes (#LC2005) for ~ 1.5 h at 30 V. Membranes were blocked in 5% milk in PBST, containing 0.1% Tween-20, for ~ 1 h, shaking at room temperature. Membranes were cut into appropriate segments and incubated in primary antibody in 5% milk in PBST overnight, at 4 °C, shaking. Primary antibody solution was washed off by 5 × 5 min washes in PBST buffer before incubation in secondary antibody in 5% milk in PBST for 1 h at room temperature, shaking. Secondary antibody solution was washed off by 5 × 5 min washes in PBST buffer.

Membranes were incubated for 2 min in Luminata Classico or Forte Western HRP substrate (Millipore, #MBLUF100) before being exposed to autoradiography film (GE Healthcare, Amersham Hyperfilm ECL, #28906837) and developed using the ECOMAX X-ray film processor (Photon Surgical Systems).

### Immunofluorescence staining

The staining protocol was slightly modified from^59^. Embryos were collected at relevant time points and fixed in 4% PFA in PBS overnight at 4 °C. Samples were washed 5 × 5 min in PBST (0.1% Tween-20), transferred into methanol and incubated for ≥ 30 min at − 20 °C. Samples were rehydrated by graded methanol washes of 5 min each, followed by 4X 5 min washes in PBST and 1X 5 min wash in PBSTX (0.1% Tween-20 and 0.1% Triton-X). Samples were blocked in 10% BSA and 1% goat serum in PBSTX for 2 h at room temperature, then incubated in primary antibody, diluted 1:200, in 1% BSA and 0.1% serum PBSTX overnight at 4 °C.

Samples were washed 6 × for 1 h in 1% BSA and 0.1% serum PBSTX and then incubated in secondary antibody, diluted 1:1,000, in the same blocking solution overnight at 4 °C. Samples were washed 6 × 5 min in PBST to remove antibody solution and immediately imaged using confocal microscopy. To prevent signal loss, samples were protected by aluminium foil from the moment of secondary antibody incubation until imaging and imaged as soon as possible.

### Image acquisition, processing, and analysis

All embryos were dechorionated and anaesthetised in 0.2% tricaine prior to imaging and mounted in 1% low-melting agarose in 35-mm-diameter glass-base dishes for confocal, or mounted in 1.5% low-melting agarose in ~ 1 mm capillaries for lightsheet imaging. All confocal images were acquired with Leica Apo 20X objective (506,147) on Leica TCS SP8, all lightsheet images were acquired with 10 × 0.5 NA W Plan-Apochromat detection and 5 × 0.1 NA illuminating objectives, with n = 1.33 optic adapter, on Zeiss Z.1. Fluorophores were excited with 488 nm or 561 nm laser lines, in confocal imaging, each *z* step was defined as 2 µm.

FIJI was used to z-project raw data into maximum intensity projections and perform measurements, for lightsheet data in particular, the pairwise stitching plug-in^60,61^ was used. For immunofluorescence imaging, GFP laser settings were determined on 1 wildtype sample and then maintained for all other samples to allow comparison of relative fluorescence intensity. For better visualisation of some features, e.g., filopodial-like protrusions, brightness and contrast were modified in FIJI. For better visualisation of the immunofluorescence staining, brightness was modified on both WT and *bcar1*^*u7000*^ image equally, by setting the max pixel value to 150 instead of 255.

Brightfield images were obtained on the OLYMPUS SZX2-ILLT/MVX10/U-HGLGPS system with OLYMPUS cellSens Standard 1.13 software. Adult fish were photographed using a Huawei P10lite mobile phone camera. Embryos and adult fish were anaesthetised lightly in 0.02% tricaine, embryos were imaged directly in the petri dish in E3, adult fish were placed onto a petri dish on top of mm paper with defined distances, then photographed and immediately returned to the tank. Scale was manually set per individual photo in FIJI before measurement. Embryonic size was measured as standard length, defined as snout to somite end, excluding the caudal fin tissue. Adult fish size was defined as snout to end of fish body, excluding caudal fin^62^.

### Statistical analysis

All experimental numbers are recorded in each figure legend, data are either presented using BoxPlotR (https://shiny.chemgrid.org/boxplotr/^63^) or visualised with GraphPad Prism version 6.07 showing mean ± 95% confidence interval. All experiments were performed on ≥ 2 independent clutches from independent parent fish. Caudal vein plexus phenotype characterisation has been performed on offspring from ≥ 3 generations of fish and has proven consistent across every transgenic background.

Data were analysed using GraphPad Prism version 6.07 software and tested for normal distribution. For comparisons of 2 groups showing normal distribution, Student’s unpaired t-test was used. If standard deviation varied between groups, Welch’s correction was applied. For sample sets not following normal distribution, the Mann–Whitney test was used. For comparisons of ≥ 2 groups, e.g., for drug inhibition studies, one-way ANOVA was used with selected pairs and Sidak’s multiple comparisons test correction was applied. Selections were either (i) comparing all groups to control or (ii) comparing inhibitor effect to control of respective genotype. Data were considered statistically significant at *p* < 0.05, *p* values are reported in each figure.

### Zebrafish husbandry

All animal experimental protocols were approved by University College London’s Animal Welfare and Ethical Review Body (AWERB) and licensed by the UK Home Office. All experiments were performed in accordance with relevant guidelines and regulations as described in UK Home Office Project License (PPL 70/8365) awarded to Dr Paul Frankel. Zebrafish were maintained in mixed-sex populations at 28 °C with a 14 h:10 h light:dark cycle from 9am-11 pm and within internationally agreed environmental parameters regarding water quality and composition. Experiments were conducted on 0–5dpf zebrafish embryos and embryos were maintained in E3 medium (5 mM NaCl, 0.17 mM KCl, 0.33 mM CaCl2 × H2O, 0.33 mM MgSO4 × 7 H2O), with addition of PTU (0.2 mM 1-phenyl 2-thiourea) if transparency was required and fish not in a transparent background.

## Supplementary information

Supplementary Information 1.

Supplementary Information 2.
