# Supplementary material for: P130Cas/bcar1 mediates zebrafish caudal vein plexus angiogenesis
Source: Sci Rep. 2020 Sep 24;10:15589. doi: 10.1038/s41598-020-71753-w (PMC7518251; doi:10.1038/s41598-020-71753-w)
Supplement: Supplementary file 1 — Supplementary Information 1. [file 41598_2020_71753_MOESM1_ESM.docx]

**Supplementary Material**

**P130Cas/*bcar1* mediates zebrafish caudal vein plexus angiogenesis.**

Laura Wisniewski2¥*, Vanessa French1, Nicola Lockwood2,3, Leonardo E. Valdivia4 and Paul Frankel1*

1. Institute of Cardiovascular Science, University College London, 5 University Street, London WC1E 6JF, UK
2. Division of Medicine, University College London, 5 University Street, London WC1E 6JF, UK
3. Current Address: The Francis Crick Institute, 1 Midland Road, London NW1 1AT, UK
4. Center for Integrative Biology, Faculty of Sciences, Universidad Mayor, Santiago, Chile

*Indicates corresponding author

¥ Current address: Queen Mary University of London, London EC1M 6BQ

Correspondence to:

Paul Frankel, email: [p.frankel@ucl.ac.uk](mailto:p.frankel@ucl.ac.uk)

Laura Wisniewski, [l.wisniewski@qmul.ac.uk](mailto:l.wisniewski@qmul.ac.uk)


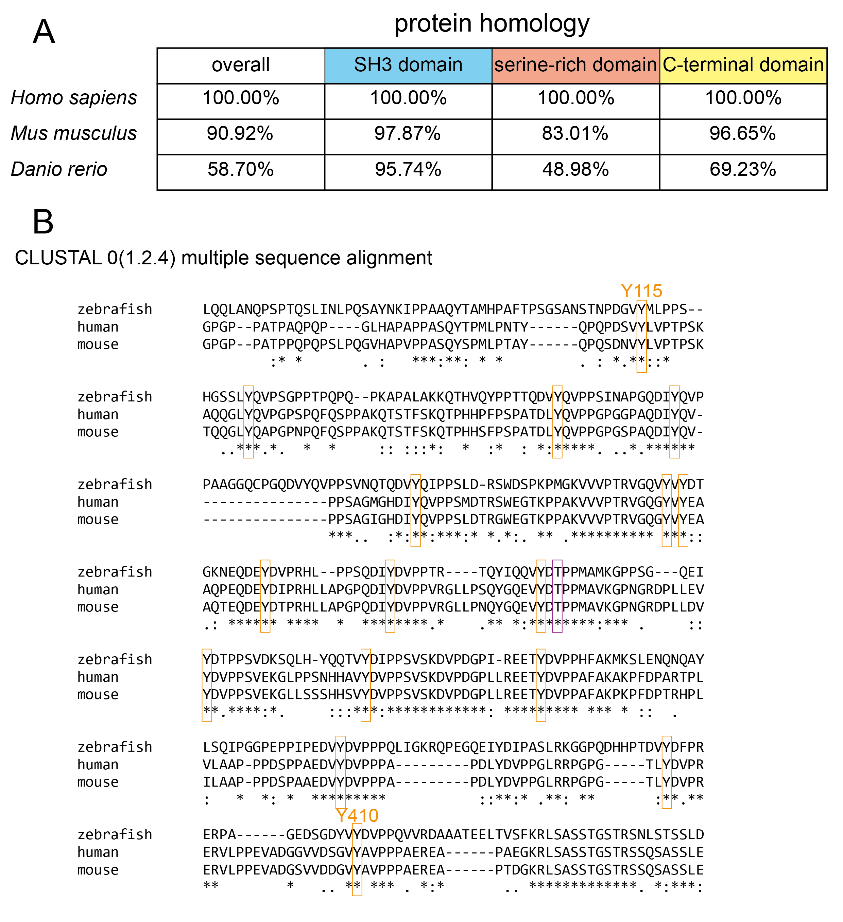


**Supplemental Figure 1: P130CAS is evolutionary conserved. (A)** Cartoon depicting human, mouse, and zebrafish P130CAS protein structure and size, with SH3 (blue), serine-rich (orange), and C-terminal CAS (yellow) domains highlighted. Protein sequence homology was calculated by the Clustal Omega software, all reference sequences were obtained from Ensembl database (accession numbers: ENSG00000050820 [Homo sapiens], ENSMUSG00000031955 [Mus musculus], ENSDARG00000056525 [Danio rerio]). **(B)** Subsection of the Clustal Omega sequence alignment in the serine-rich substrate binding domain. Tyrosine (Y) residues important for P130CAS activation and signalling are highlighted by orange boxes. Note that all tyrosine residues are fully conserved.

*
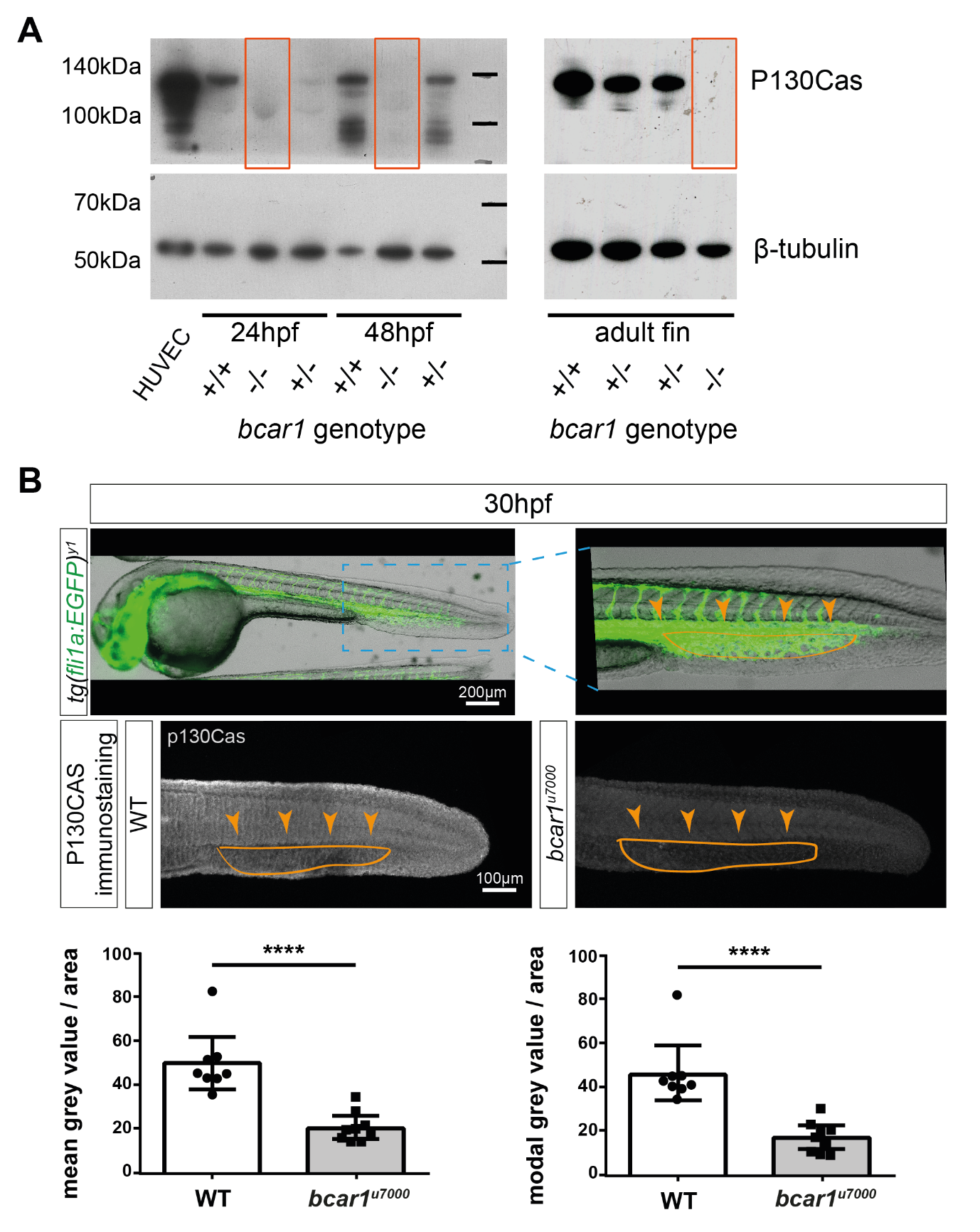
*

**Supplemental Figure 2: P130Cas is expressed during embryonic development and absent in bcar1^u7000^ mutant zebrafish. (A)** Immunoblotting shows that P130Cas protein is detected in zebrafish embryos and adult fin tissue, using HUVEC cell lysate as antibody binding control. P130Cas detection is not possible in bcar1^u7000^ samples (indicated by orange boxes), indicating permanent loss of protein. Blots shown are representative of ≥ 4 independent sample sets. **(B)** Brightfield-GFP overlay as area clarification (top) and maximum intensity projection of P130Cas immunofluorescence staining (middle) in WT and bcar1^u7000^ embryos. Quantification graphs (bottom) show highly significant loss of P130Cas signal in bcar1^u7000^ embryos, further confirming loss of protein. Images are representative of n=8 wildtype and n=9 bcar1^u7000^ embryos, stained independently n=3 times, Mann-Whitney test, p<0.0001. Scale bars as indicated.


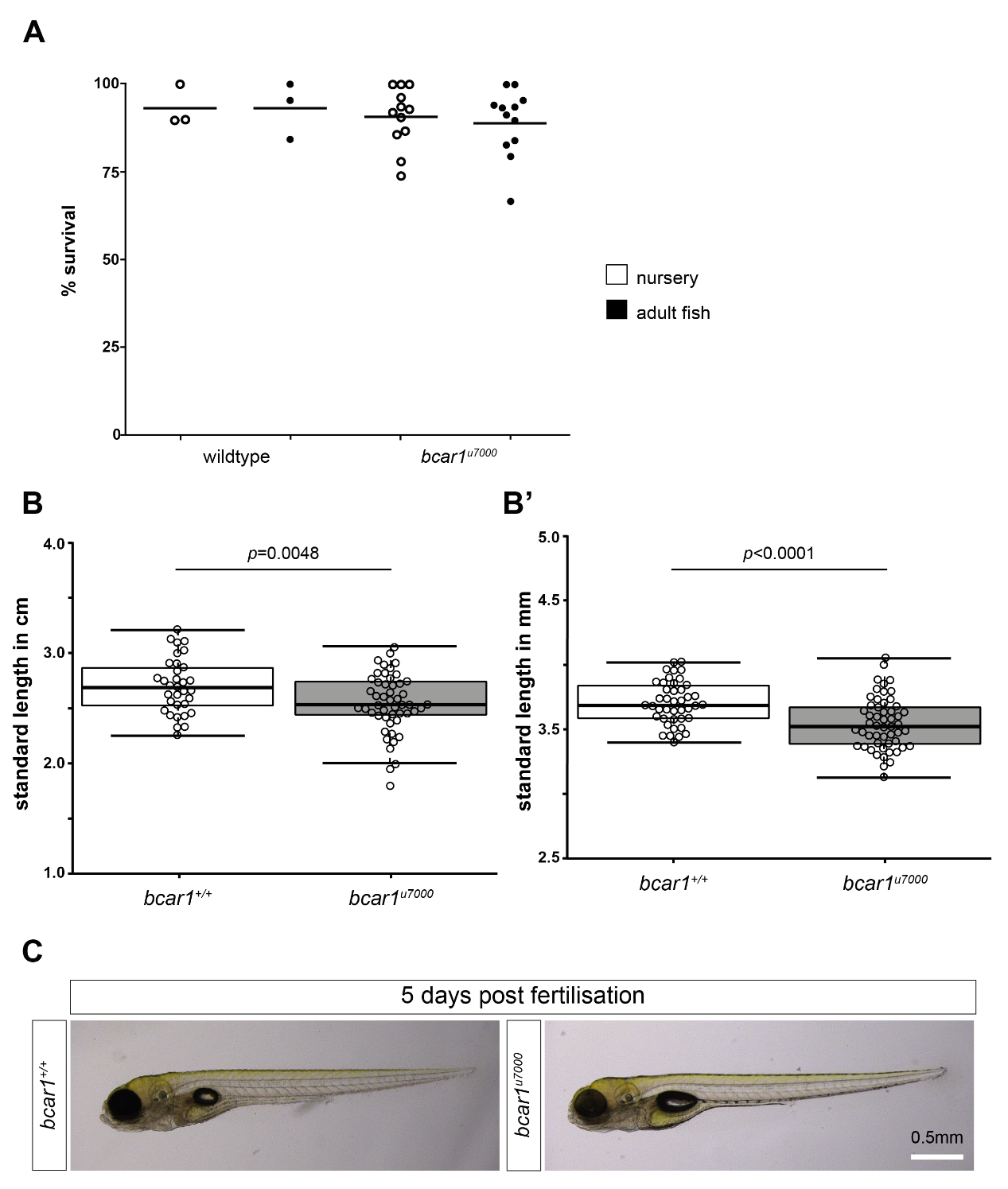


**Supplemental Figure 3: Loss of P130Cas does not affect viability but causes a small reduction in fish size. (A)** Bcar1^u7000^ fry survive with average rates during nursery rearing (=until 56dpf, as monitored by facility staff) and throughout adulthood (recorded until ~12-15 months post fertilisation, n=455 fish, each dot represents overall survival of a generation). **(B, B’)** Adult fish **(B)** and bcar1^u7000^ embryos at 5dpf **(B’)** show a subtle, but statistically significant reduction in standard length. N=51 WT and n=34 bcar1^u7000^ adults, n=42 WT and n=53 bcar1^u7000^ embryos analysed, from n=3 independent generations/clutches. Unpaired t-test, p values as indicated. **(C)** Brightfield images of WT and bcar1^u7000^ embryos at 5dpf showing that morphologically, bcar1^u7000^ mutant fish appear indistinguishable from WT siblings at 5dpf or in adulthood (see figure 1B for photographs of adult fish). Mutants do not display overt defects such as skeletal deformations, lack of swim bladder or somite defects. Scale bar as indicated.


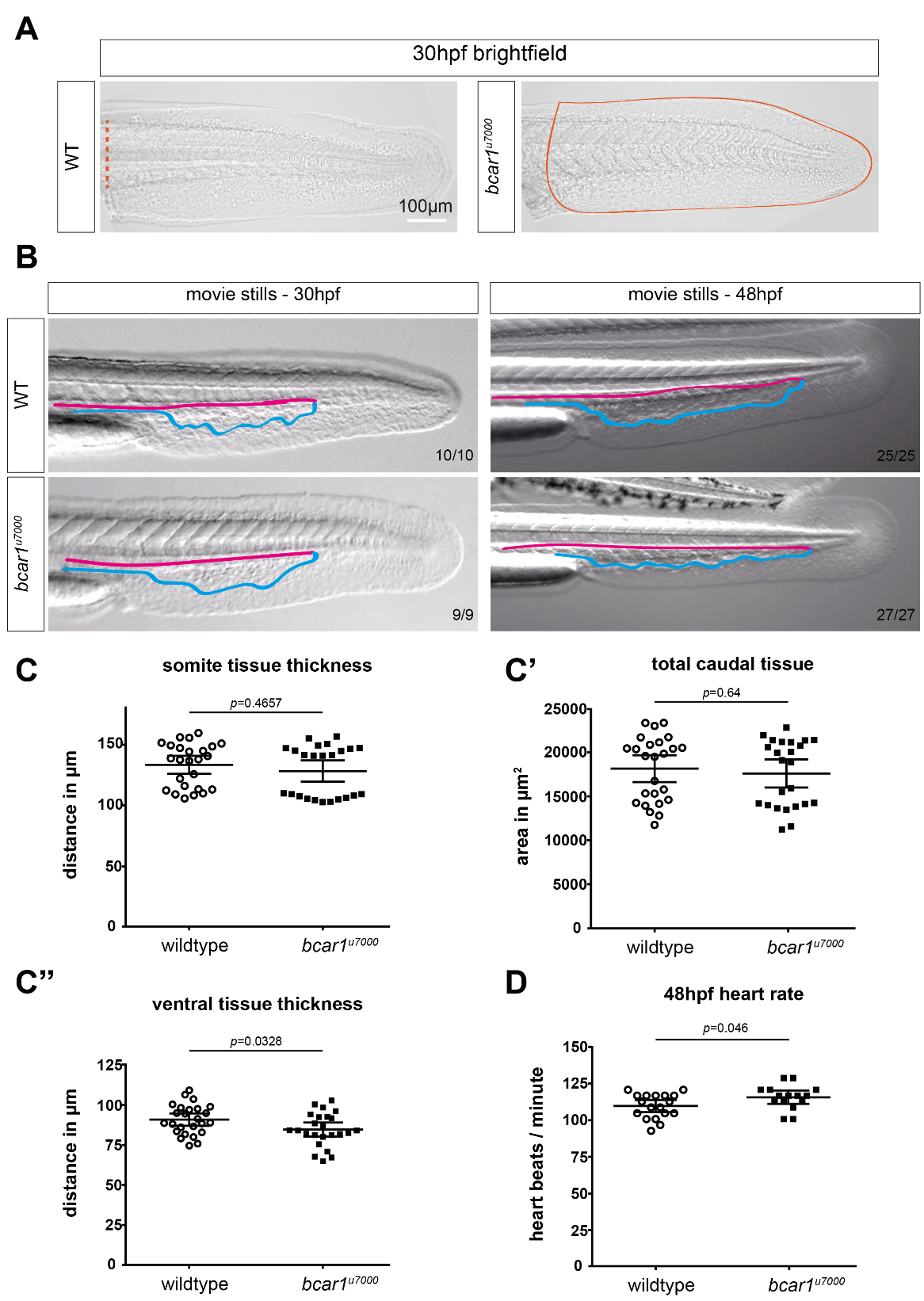


**Supplemental Figure 4: P130Cas is not required for overall caudal tissue formation or gross blood flow regulation. (A)** Brightfield images showing the caudal tissue of WT and bcar1^u7000^ embryos at 30hpf. **(B)** Brightfield movie stills of the caudal tissue of WT and bcar1^u7000^ embryos at 30hpf (left) and 48hpf (right). The visually observed blood flow pattern is indicated in magenta and blue, for arterial and venous flow, respectively. No overt differences in flow velocity were observed. N as indicated. **(C-C’’)** Quantification of somite tissue thickness **(C)** (as measured at anal pore, indicated by orange dotted line in A, left) and total caudal tissue area **(C’)** (from anal pore, indicated by orange line drawing in A, right) shows no difference between WT and bcar1^u7000^, Mann-Whitney test, p=0.47 and p=0.64 respectively. The ventral tissue thickness **(C’’)** is slightly reduced in bcar1^u7000^ embryos, Student’s t-test, p=0.0328. N=25 WT and n=24 bcar1^u7000^, >4 clutches. **(D)** At 48hpf, the heart rate of bcar1^u7000^ embryos is modestly increased, n=18 WT and n=15 bcar1^u7000^ embryos, Student’s t-test, p=0.046. All graphs show mean ± 95% confidence interval. Scale bars as indicated.


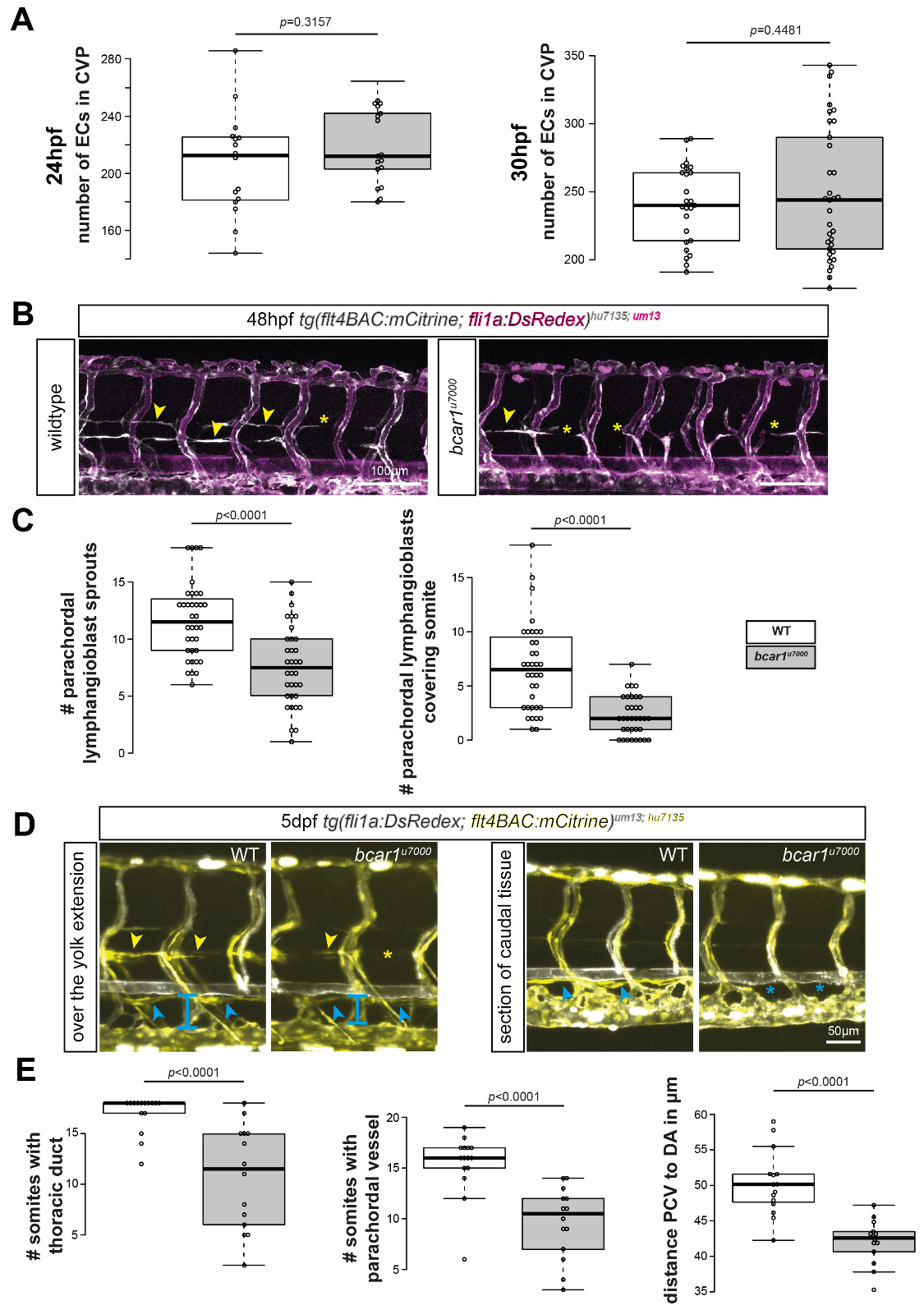


**Supplemental Figure 5: P130Cas is not required for EC proliferation but promotes lymphangiogenesis in the trunk. (A)** Quantification of EC numbers using tg(fli1a:nEGFP)^y7^ embryos at 24hpf (left) and 30hpf (right) showed no difference between WT and bcar1^u7000^ embryos. **(B, D)** Maximum intensity projection images of trunk sections of wildtype and bcar1^u7000^ embryos at 48hpf **(B)** and 5dpf **(D)**. Arrowheads indicate parachordal lymphangioblasts (PLs) in (B) or thoracic duct and parachordal vessel in **(D)**. Blue line indicates the distance between dorsal aorta and cardinal vein over the yolk extension **(D left)**. **(C, E)** Quantification of PL number showed significantly fewer PLs emerging and fewer PLs stretching across the somite **(C)**, as well as significantly fewer somites which showed thoracic duct (left) or parachordal vessel (middle)in bcar1^u7000^ embryos **(E)**. Over the yolk extension, the cardinal vein remained significantly closer to the dorsal aorta. All results displayed as box plots with centre line showing median and box dimensions indicating 25^th^ and 75^th^ quartile. Each data point represents an individual embryo, from n ≥ 3 independent clutches. White boxes are wildtype, light grey boxes indicate bcar1^u7000^. Scale bars as indicated.

**
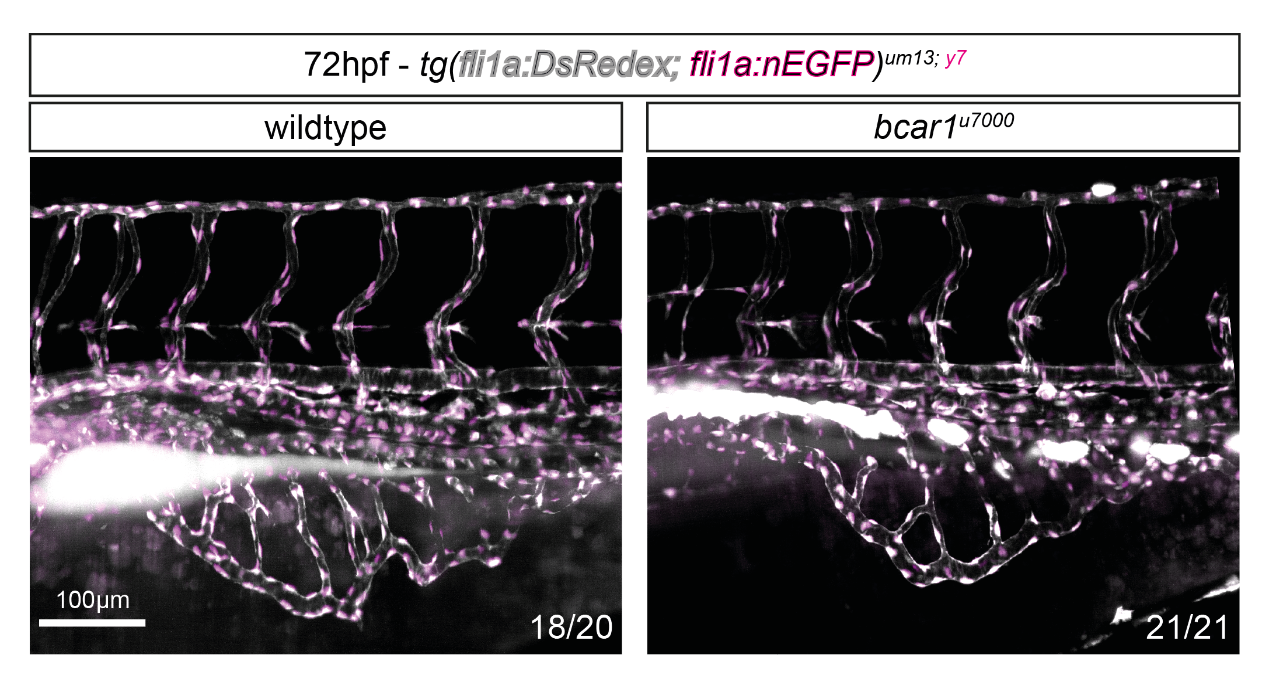
**

**Supplemental Figure 6: P130Cas is not required for formation of the subintestinal vein plexus.** Maximum intensity projections of wildtype and bcar1^u7000^ embryos at 72hpf show the characteristic basket structure of the subintestinal vein plexus, covering the yolk, suggesting that P130Cas is not required for angiogenesis in this vascular bed. Experimental n numbers are indicated in each image, scale bar as indicated.
