# Supplementary material for: P130Cas/bcar1 mediates zebrafish caudal vein plexus angiogenesis
Source: Sci Rep. 2020 Sep 24;10:15589. doi: 10.1038/s41598-020-71753-w (PMC7518251; doi:10.1038/s41598-020-71753-w)

P130Cas/*bcar1* mediates zebrafish caudal vein plexus angiogenesis.

Laura Wisniewski1¥*, Vanessa French1, Nicola Lockwood1,2, Leonardo E. Valdivia3 and Paul Frankel1*

1) Division of Medicine, University College London, 5 University Street, London WC1E 6JF, UK
2) Current Address: The Francis Crick Institute, 1 Midland Road, London NW1 1AT, UK
3) Center for Integrative Biology, Faculty of Sciences, Universidad Mayor, Santiago, Chile

*Indicates corresponding author

¥ Current address: Queen Mary University of London, London EC1M 6BQ

Correspondence to:

Paul Frankel, email: p.frankel@ucl.ac.uk

Laura Wisniewski, l.wisniewski@qmul.ac.uk

Supplementary Information File

Full-length blots used in Supplemental Figure 2A.

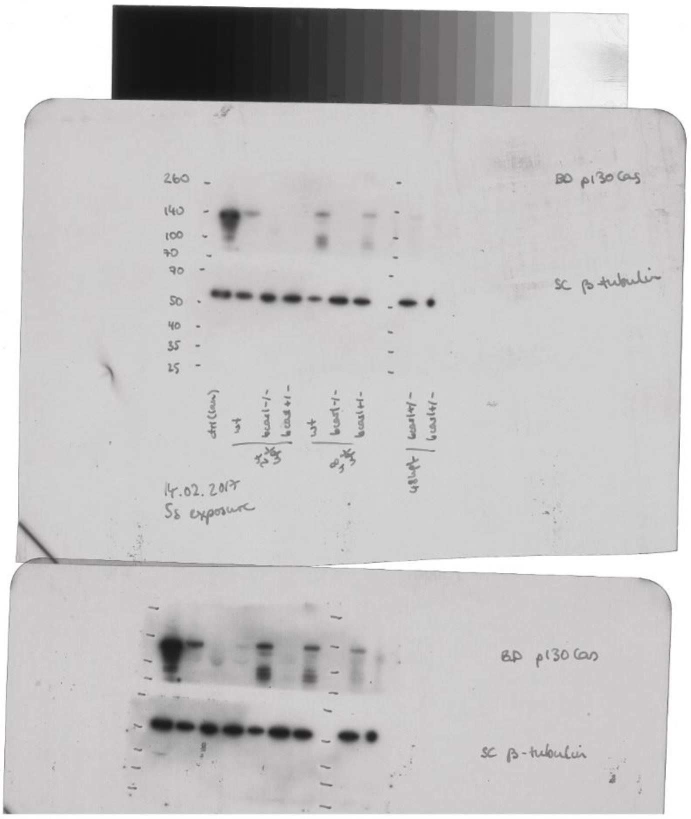

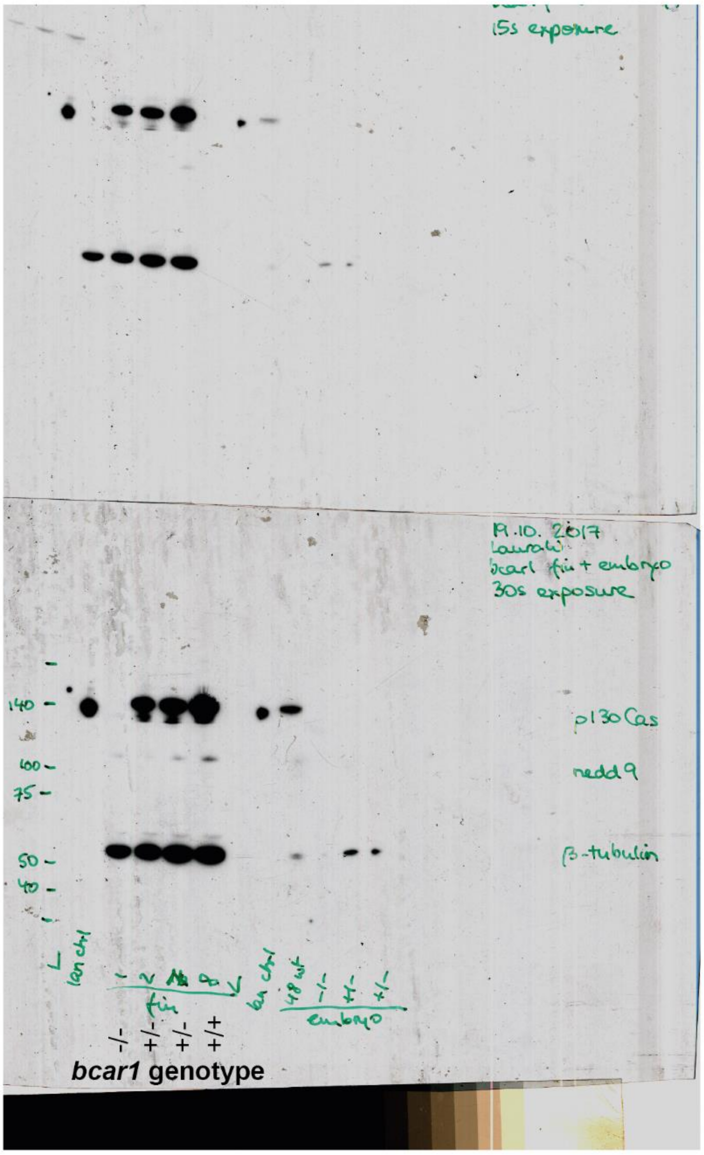

Author comment on blot orientation

In Supplemental Figure 2A, the blot showing protein lysates derived from adult zebrafish fin tissue is shown horizontally flipped to the original scan (shown here on the right hand side). The genotypes of the samples are indicated by overlaid writing at the bottom of the scan.

The flip was performed to keep the order of samples' genotypes consistent within the panel, i.e., wildtype genotyped samples before heterozygous or homozygous genotyped samples.

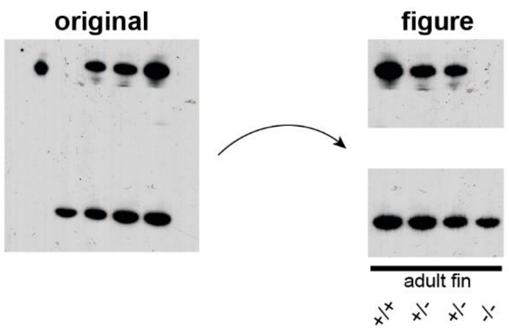

Supplement: Supplementary file 2 — Supplementary Information 2. [file 41598_2020_71753_MOESM2_ESM.pdf]
